# The *TLR9* Gene Polymorphisms and the Risk of Cancer: Evidence from a Meta-Analysis

**DOI:** 10.1371/journal.pone.0071785

**Published:** 2013-08-19

**Authors:** LuShun Zhang, HaoJie Qin, Xuan Guan, Kui Zhang, ZhiRong Liu

**Affiliations:** 1 Department of Pathology, Cheng Du Medical College, Chengdu, China; 2 School of Forensic Medicine, Henan University of Science and Technology, Luoyang, China; 3 Department of Forensic Medicine, Zun Yi Medical College, Zunyi, China; 4 Department of Breast and Vascular Surgery, Second People’s Hospital of Chengdu, Chengdu, China; University of Aberdeen, United Kingdom

## Abstract

**Background:**

Growing studies have revealed the association between polymorphisms in the Toll-like receptor 9 (*TLR9*) and susceptibility to cancer, however, the results remained inconsistent.

**Methodology/Principal Findings:**

To assess the effect of three selected SNPs (rs352140, rs5743836 and rs187084) in *TLR9* on cancer, we performed a meta-analysis based on 11 case-control studies, including a total of 6,585 cancer cases and 7,506 controls. Summary odds ratios (OR) and corresponding 95% confidence intervals (CIs) for polymorphisms in *TLR9* and cancer risk were estimated. Our meta-analysis indicated that rs352140 was associated with an increased cancer risk, especially in Caucasian. However, no significantly increased cancer risk was detected to be associated with rs187084 and rs5743836 either the overall or subgroup estimation.

**Conclusions:**

These meta-analysis results indicate that polymorphisms in *TLR9* may play a role in cancer development.

## Introduction

Toll-like receptors (TLRs), expressed predominantly on antigen-presenting cells, belongs to the family of pattern-recognition receptors (PRRs). In humans, TLR family consists of 10 members (TLRs 1–10) [1]. It plays an essential role in immune response against microbial pathogens by recognizing specific microbial molecular components. After activated, TLRs initiate a signaling cascade resulting in the stimulation of innate and adaptive immune responses targeting the invading pathogen [2], [3]. Although TLRs have been implicated as the first line defense in human for anti-microbial responses, they also participate in the pathophysiology of many inflammatory and immune diseases, including cancer [4]–[7].

Human *TLR9* is located on chromosome 3p21.3 [8], contains two exons and is preferentially expressed by B cells and plasmacytoid dendritic cells [9]. Unlike other members of the TLR gene family that constitute the membrane-bound pattern recognition receptors, TLR9 is localized on the endoplasmic reticulum membrane (in the resting state) or on the endosomal/lysosomal membrane (after ligand stimulation and trafficking) [10], [11]. TLR9 recognizes unmethylated CpG motifs present in bacteria and viruses [12]. Alternatively, TLR9 functions through the myeloid differentiation primary response protein 88 (MyD88)-dependent pathway leading to NF-kappa-B (NF-kB) activation, cytokine secretion and the inflammatory response [12], [13]. In the past years, numerous genetic association studies have explored the role of *TLR9* gene polymorphisms in various cancers, including bladder cancer [14], prostate cancer [15], acute lymphoblastic leukemia (ALL) [16], hepatocellular carcinoma (HCC) [17], gastric cancer [18]–[20], cervical cancer [2], [13], [21], Hodgkin’s lymphoma [22], breast cancer [23], burkitt’s lymphoma [24], non-Hodgkin lymphoma [25], endometrial cancer [26], esophageal cancer [20] and lymphoma [27]. Most of the studies focused on three common single nucleotide polymorphisms (SNPs), including rs352140(C/T), rs5743836 (T/C) and rs187084(C/T) (also referred to as 2848C/T, 1237T/C, and 1486C/T, respectively). However, the results remained inconsistent.

Considering a single study might underpowered to detect the overall effects in complex diseases, a quantitative synthesis of the accumulated data from different studies was deemed important to provide evidence on the association of variants in *TLR9* with cancer risk. Thus, we performed this meta-analysis with accumulated data to evaluate the overall cancer risk of selected three SNPs in *TLR9* and to quantify heterogeneity between the individual studies as well as to investigate the existence of potential publication bias.

## Materials and Methods

### Search Strategy

We searched PubMed and CNKI (China National Knowledge Infrastructure) for all articles on the association between *TLR9* polymorphisms and Cancer risk (updated to January 20, 2013) using the following terms: ‘Toll like receptor 9’ or ‘*TLR9*’ and ‘cancer’ or ‘tumor’ or ‘carcinoma’ and ‘polymorphism’ or ‘polymorphisms’ or ‘SNP’ for relevant reports. In order to identify the relevant publications, the references cited in the research papers were also scanned.

### Inclusion and Exclusion Criteria

The inclusion criteria for current meta-analysis studies were (1) evaluation of the *TLR9* polymorphism and cancer risk, (2) being a case-control study, (3) existing useful genotype frequency (or data available to calculate them), (4) control subjects satisfied the Hardy-Weinberg equilibrium (HWE), and (5) the study was published in English or Chinese. Abstracts and unpublished reports were not considered.

### Data Extraction

Data extraction was independently done by two investigators (Zhang L.S. and Qin H.J.), and discrepancies were resolved by consensus including a third investigator (Zhang K.). The following characteristics were collected from each study: first author, year of publication, country of the study population, ethnicity, cancer types, genotyping methods, number of genotypes, sample size, minor allele frequency (MAF) in controls, and evidence of Hardy-Weinberg equilibrium (HWE). Eligible studies were stratified into population-based (PB) and hospital-based (HB) according to the control source. Once studies include subjects of different ethnic groups, data were extracted separately for each ethnic group.

### Methodological Quality Assessment

The quality of eligible studies was evaluated by three reviewers (Zhang L.S., Guan X. and Qin H.J.) independently by scoring according to a ‘methodological quality assessment scale’ (Table S2), which was referred to previous meta-analysis [28], [29]. In the scale, five items were assessed, including namely the representativeness of cases, source of controls, ascertainment of relevant cancer, sample size and quality control of genotyping methods. Quality scores ranged from 0 to 9 and a high score indicated good quality of the study. Only studies with a score of 6 or higher were included.

### Statistical Analysis

HWE in controls for each study was assessed by using a goodness of fit chi-square test before statistical analysis and *P*<0.05 was considered as significant disequilibrium. Strength of association between *TLR9* polymorphism and cancer risk was evaluated by crude odds ratio (OR) and 95% confidence interval (CI). Pairwise group differences of ORs were analyzed and the best genetic models were to be determined according to the Thakkinstian’s method [30]. Data were then pooled using the best model. Ethnicity, cancer types and sample size were adopted to carry out the stratified analysis, when data were available.

A chi-square-based Q test was used to check the statistical heterogeneity [31]. If the result of the heterogeneity test was *P*>0.1, then ORs were pooled according to the fixed-effects model (the Mantel-Haenszel model) [32]. Otherwise, the random effects model (the DerSimonian-Laird model) was used [33]. To explore sources of heterogeneity across studies, we did logistic meta-regression analysis, and assessed all comparison models by frequency of minor allele in control subjects, frequency of minor allele in case subjects, cancer types, the source of control, sample size and ethnicity. Publication bias was checked using the Begg test [34] and the Egger test [35]. Sensitivity analysis was performed to assess the stability of the result with each study in turn being removed. All statistical tests were performed with the software STATA version 11.0 (Stata Corporation, College station, TX).

## Results

### Characteristics of Studies

**Figure 1:**
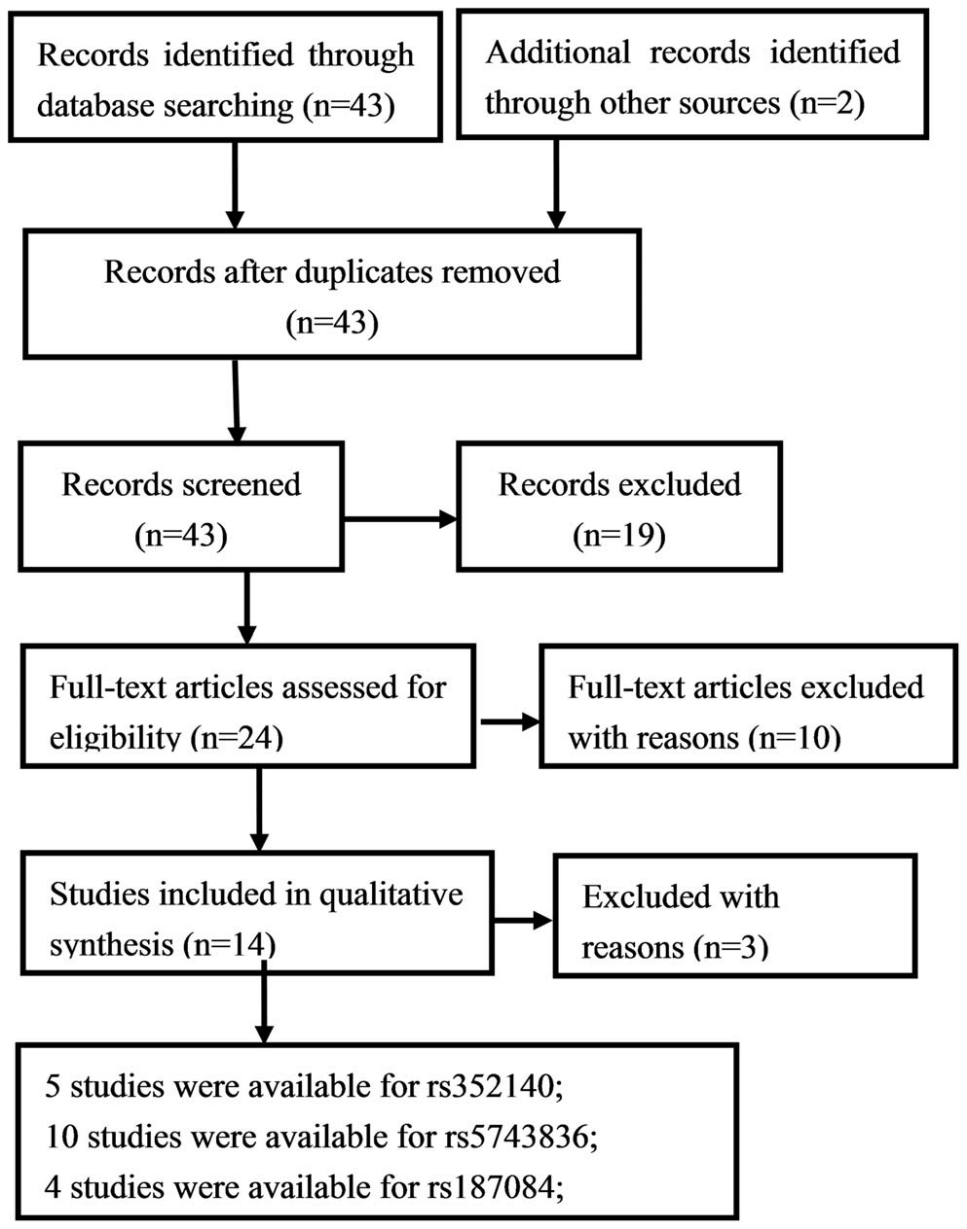
Flowchart for identification of studies.

Our initial search identified 43 studies according to the search words, and 2 records added through the reference scan. After screening the abstract, 24 were retrieved for more detailed evaluation. After reviewing full text, 10 studies were excluded for the following reasons, three papers were reviews [5], [11], [36], two papers were relating to risk of infection during cancer therapy [37], [38], one paper was relating to outcome of AML patients transplanted from HLA-identical sibling donors [39], two papers lacked of gene frequency data to calculate the ORs and 95% CI [40], [41], and two of the studies were disequilibrium from HWE in control group [13], [15]. One article evaluated two SNPs (rs352140 and rs5743836) with Burkitt’s lymphoma risk [24], and genotype distribution in the control of rs352140 was inconsistent with HWE (P<0.05). Therefore, we extracted the data relating to rs5743836 for our meta-analysis. Only one study evaluated rs352139 polymorphism and cancer susceptibility [17]. Thus, the data were not available for the meta-analysis. The detailed screening process was shown in Figure 1. The results of ‘methodological quality assessment scale’ showed that the quality scores ranged from 5.5 to 8, and 3 studies were excluded for the score less than 6 [18], [19], [21]. Finally, 11 case–control studies contain three separate SNPs (rs352140, rs5743836 and rs187084) were selected for this meta-analysis. Study characteristics were summarized in Table 1. As shown in table 1, five studies were available for rs352140 [14], [16], [17], [22], [23], ten studies were available for rs5743836 [16], [20], [22], [24]–[27], and four studies were available for rs187084 [2], [16], [26], [27]. Those include studies in Germany, Poland, Netherlands, Greece, Croatia, Portugal, USA, Australia, India and China. Several genotyping methods were used, including polymerase chain reaction - restriction fragment length polymorphism (PCR-RFLP) assay, TaqMan probe, Competitive Allele-Specific PCR (ASPCR), bi-directional PCR amplification of specific alleles (Bi-PASA), tetra-primer assays and multiplex polymerase chain reactions SNaPshot method (Multiplex PCR SNaPshot).

**Table 1:** Characteristics of studies included in the meta-analysis.

Reference	Year	Region	Ethnic	Genotypingmethod	Cancer type	Control	Genotype-case			Genotype-control			MAF	HWE
rs352140							CC	CT	TT	CC	CT	TT		
Singh, V.	2012	Indian	Asian	PCR-RFLP	Bladder cancer	HB	96	89	15	83	97	20	0.658	Y
Miedema, K.G.	2012	Netherlands	Caucasian	ASPCR	ALL	PB	31	85	67	36	88	57	0.442	Y
Junjie, X.	2012	China	Asian	Multiplex PCR SNaPshot	HCC	HB	96	85	30	102	109	21	0.675	Y
Mollaki, V.	2009	Greece	Caucasian	PCR-RFLP	Hodgkin’s lymphoma	HB	2	49	39	11	50	31	0.391	Y
Etokebe, G.E.	2009	Croatia	Caucasian	TaqMan	Breast cancer	HB	45	60	25	36	49	12	0.624	Y
**rs187084**							**TT**	**CT**	**CC**	**TT**	**CT**	**CC**		
Chen, X.	2012	China	Asian	PCR-RFLP	Cervical cancer	PB	246	346	102	289	319	107	0.373	Y
Ashton, K.A.	2010	Australia	Caucasian	TaqMan	Endometrial cancer	PB	138	49	4	187	88	16	0.206	Y
Miedema, K.G.	2012	Netherlands	Caucasian	ASPCR	ALL	PB	57	88	37	50	98	31	0.447	Y
Nieters, A.	2006	Germany	Caucasian	PCR-RFLP	Lymphoma	PB	227	332	117	230	331	106	0.407	Y
**rs5743836**							**TT**	**CT**	**CC**	**TT**	**CT**	**CC**		
Noack, J.	2012	Germany	Caucasian	tetra-primer assays	Burkitt’s lymphoma	PB	11	4	0	343	61	0	0.075	Y
Carvalho, A.	2012	Portugal	Caucasian	Bi-PASA	Non-Hodgkin lymphoma	HB	551	244	2	934	217	9	0.101	Y
Carvalho, A.	2012	Italy	Caucasian	Bi-PASA	Non-Hodgkin lymphoma	HB	345	135	14	379	81	8	0.104	Y
Carvalho, A.	2012	USA	Caucasian	TaqMan	Non-Hodgkin lymphoma	HB	579	209	13	674	275	23	0.165	Y
Mollaki, V.	2009	Greece	Caucasian	PCR-RFLP	Hodgkin’s lymphoma	HB	39	50	1	61	31	0	0.168	Y
Miedema, K.G.	2012	Netherlands	Caucasian	ASPCR	ALL	PB	133	45	7	138	40	4	0.132	Y
Ashton, K.A.	2010	Australia	Caucasian	TaqMan	Endometrial cancer	PB	85	79	27	116	128	47	0.381	Y
Hold, G.L.	2009	USA	Caucasian	Taqman	Esophageal Cancer/Gastric cancer	PB	341	98	12	149	57	4	0.155	Y
Hold, G.L.	2009	Poland	Caucasian	Taqman	Gastric cancer	PB	261	58	7	316	85	5	0.117	Y
Nieters, A.	2006	German	Caucasian	PCR-RFLP	Lymphoma	PB	507	156	15	475	181	11	0.152	Y

MAF: minor allele frequency in controls, HWE: Hardy-Weinberg equilibrium in controls, OR: odds ratio estimates, CI: confidence interval.

PCR-RFLP: polymerase chain reaction - restriction fragment length polymorphism, Competitive ASPCR: Allele-Specific PCR, Bi-PASA: bi-directional PCR amplification of specific alleles, Multiplex PCR SNaPshot: multiplex polymerase chain reactions SNaPshot method, PB: population-based, HB: hospital-based.

ALL: acute lymphoblastic leukemia, HCC: hepatocellular carcinoma.

### Quantitative Synthesis

**Figure 2:**
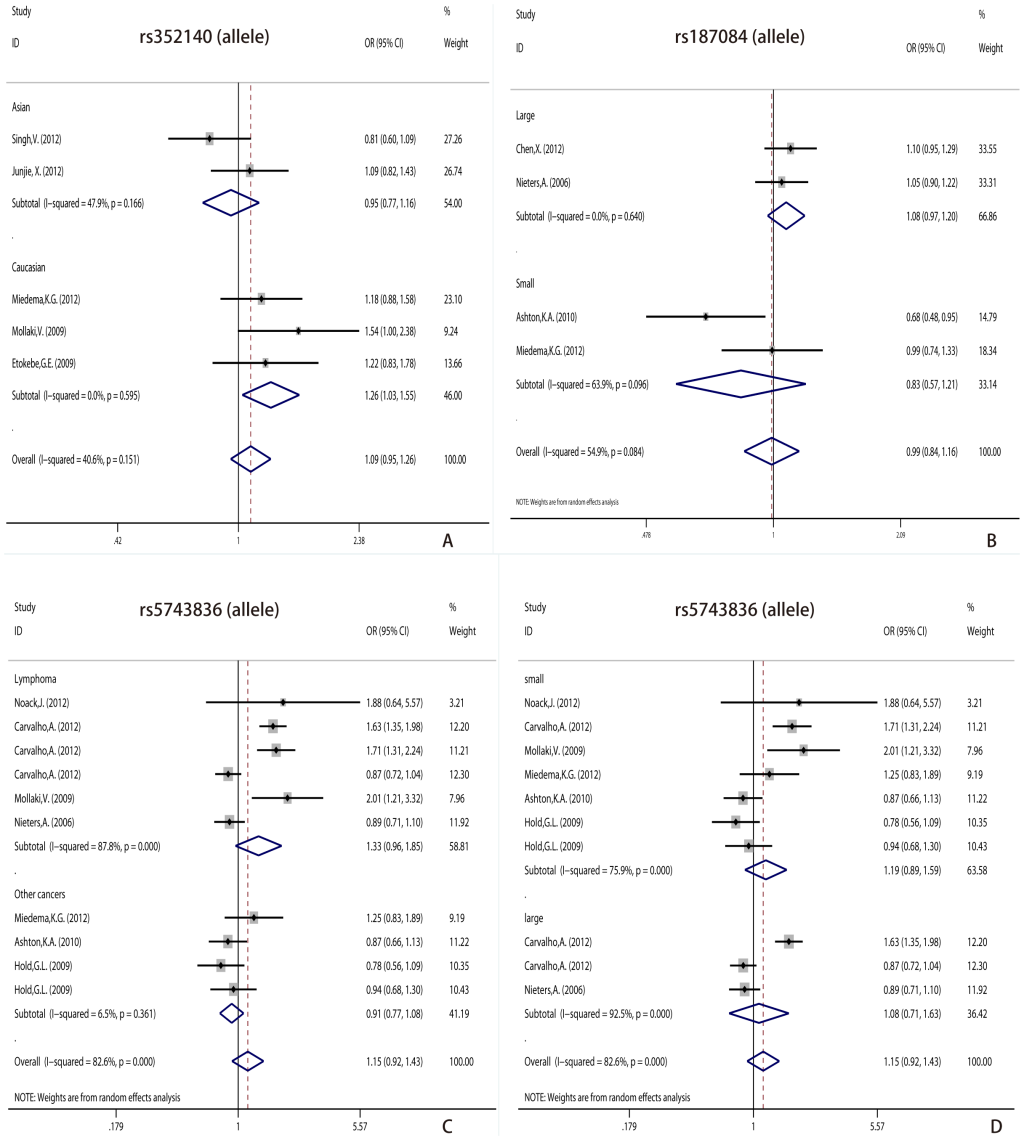
Forest plot of cancer risk associated with variants of *TLR9*. A. Comparison of the *TLR9* rs352140 polymorphism allele comparison (T allele vs. C allele) with cancer risk in subgroup of ethnicity under the fixed-effects model. B. Comparison of the *TLR9* rs187084 polymorphism allele comparison (C allele vs. T allele) with cancer risk in subgroup of sample size under the random-effects model. C. Comparison of the *TLR9* rs5743836 polymorphism allele comparison (C allele vs. T allele) with cancer risk in subgroup of cancer types under the random-effects model. D. Comparison of the *TLR9* rs5743836 polymorphism allele comparison (C allele vs. T allele) with cancer risk in subgroup of sample size under the random-effects model.

For control subjects, the MAF ranges from 0.39 to 0.66 in rs352140, from 0.08 to 0.38 in rs5743836 and from 0.21 to 0.45 in rs187084. Overall, for rs352140, rs5743836 and rs187084, no significant difference between cancers and controls were detected in allele comparison (Figure 2, Table S4).

For rs352140, the estimated OR1 (TT versus CC), OR2 (CT versus CC) and OR3 (TT versus CT) were 1.414 (95% CI: 0.854, 2.342), 0.937 (95% CI: 0.746, 1.178) and 1.309 (95% CI: 0.996, 1.721), and all of them were not significant (*P* values were 0.178, 0.577 and 0.053, respectively) (Table S3). Thus, we mainly pooled OR for allele comparison and recessive genetic model in the subgroup analysis by ethnicity. The pooled examination displayed that allele T genotype TT were increase susceptibility to cancer risk (T/C: OR = 1.263, 95% CI: 1.029, 1.551, *P* = 0.026, *P* ^heterogeneity^ = 0.595; TT vs. CC/CT: OR = 1.397, 95% CI: 1.017, 1.919, *P* = 0.039, *P* ^heterogeneity^ = 0.766) among Caucasian, but not in Asian (Table S4).

Similarly, for rs187084, the estimated OR1 (TT versus CC), OR2 (CT versus CC) and OR3 (TT versus CT) were 1.054 (95% CI: 0.857, 1.296), 0.992 (95% CI: 0.784, 1.255) and 1.008 (95% CI: 0.797, 1.276), and all of them were not significant (*P* values were 0.621, 0.946 and 0.971, respectively) (Table S3). So, we mainly pooled OR for allele comparison and recessive genetic model in the subgroup analysis by sample size (studies with more than 1000 participants were categorized as ‘large’, and studies with less 1000 participants were categorized as ‘small’). However, no significant difference between cancers and controls were detected in the subgroup analysis by sample size (Figure 2, Table S4).

Due to the missing of the minor allele in some studies, it was difficult to get the OR1, OR2, and OR3. Hence, we performed the allele comparison and dominant comparison to evaluate the association between rs187084 polymorphism and cancer risk. However, we did not found any significant association between this SNP and the cancer risk in subgroup analysis by cancer types (lymphoma and other cancers) (Figure 2, Table S4).

### Heterogeneity Analysis

Heterogeneity was detected among studies in overall comparisons (C vs. T: I^2^ = 54.9%, *P* ^heterogeneity^ = 0.084, dominant model: I^2^ = 58.9%, *P* ^heterogeneity^ = 0.063 for rs187084; C vs. T: I^2^ = 82.6%, *P* ^heterogeneity^<0.001, dominant model: I^2^ = 85.1%, *P* ^heterogeneity^<0.001 for rs5743836; respectively) and subgroup analysis. To explore sources of heterogeneity across studies, we assessed allele comparison by frequency of minor allele in control subjects, frequency of minor allele in case subjects, cancer types, ethnicity and sample size, when it was available. As a result, frequency of minor allele in control subjects of rs187084 could explain 82.35% of the heterogeneity. Frequency of minor allele in control subjects of rs5743836 and cancer types could explain a total proportion of 62.2% to the heterogeneity.

### Sensitivity Analysis and Publication Bias

To evaluate the effect of individual study on the overall meta-analysis estimate, we excluded one study at a time, and the omission of any single study made no significant difference, suggesting that the results of this meta-analysis were stable.

**Figure 3:**
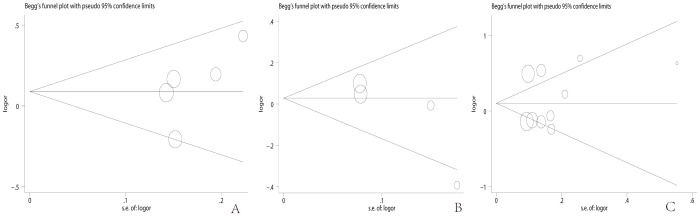
Begg’s funnel plot for overall publication bias test. A: *TLR9* rs352140, B, *TLR9* rs187084, C *TLR9* rs5743836. Each point represents a separate study for the indicated association. Log OR, natural logarithm of OR; s.e., standard error; horizontal line, mean effect size.

Begg’s funnel plot and Egger’s test were performed to assess publication bias of the selected literature. No evidence of publication bias in our study was observed (Begg’s test *P* = 0.221, Egger’s test *P* = 0.237 for rs352140; Begg’s test *P* = 0.089, Egger’s test *P* = 0.155 for rs187084; Begg’s test *P* = 0.283, Egger’s test *P* = 0.613 for rs5743836; respectively) (Figure 3).

## Discussion

In this study, we performed a meta-analysis to explore the association between *TLR9* polymorphisms and cancer risk among 14,091 subjects. Our meta-analysis identified that elevated cancer risk was statistically associated with TT genotype in recessive model of rs352140. Moreover, in terms of stratified analysis by ethnicity, allele T and genotype TT were found to increase cancer risk in both allele comparison and recessive model among Caucasian. However, no significant associations were found between rs187084 and rs5743836 polymorphisms and cancer risk either in the overall comparison or in subgroup analysis.

The synonymous rs352140 polymorphism locates in exon 2 of *TLR9*. Previous research stated that rs352140 TT genotype was associated with a higher expression of *TLR9* at the mRNA level [21], [42] and increased frequency of IgM+B cells [42]. Increased expression of the rs352140 T variant in precursor malignant lesion cells combined with infection by various pathogens might support inflammation and cervical cancer development [5], [21]. It has been believed that chronic inflammation may lead to cancer development and progression [5]. Therefore, rs352140 might affect the innate immune response, inflammation and subsequent carcinogenesis. During the last few years, numerous studies were undertaken to assess the association between rs352140 polymorphism and cancer processes, but the results were inconsistent. Some studies showed an association between rs352140 polymorphism and several types cancer progress, including Hodgkin’s lymphoma [22], Burkitt’s lymphoma [24] and cervical cancer [21] in Caucasians. However, studies performed in bladder cancer [14], prostate cancer [15], hepatocellular carcinoma [17], gastric cancer [19] and cervical cancer [13] found no association with the rs352140 polymorphism among Asian population. Similar negative results were observed in acute lymphoblastic leukemia [16] and breast cancer [23] in Caucasian population. After analysis the HWE in those studies which evaluated rs352140 polymorphism association with the cancer risk, we found three studies were disequilibrium from HWE in control subjects [13], [15], [24]. Deviation from HWE may be due to genetic reasons including non-random mating, or the alleles reflect recent mutations that have not reached equilibrium, as well as methodological reasons including biased selection of subjects from the population or genotyping errors [43]–[45]. So, we drop those three studies in present meta-analysis. We also drop the data of Zhang L [19], Zeng HM. et al. [18] and Roszak, A. et al. [21] for their low quality in assessment score due to no total description about the source of controls. In addition, former studies indicated that if the tests were not performed properly, the total false positive rate, for the four genetic models, increases to 0.23 if the prior type one error is 0.05 [30], [46]. In this meta-analysis, we did not find an appropriate genetic model according to the Thakkinstian’s method [30]. Therefore, we chose allele comparison and recessive genetic model to evaluate the association between the polymorphism and the cancer risk in the overall and subgroup. We observed that elevated cancer risk was statistically associated with TT genotype in recessive model of rs352140. Moreover, in terms of stratified analysis by ethnicity, allele T and genotype TT were found to increase cancer risk in allele comparison and recessive model among Caucasians. Former studies revealed that rs352140 polymorphism might influence infection during cancer development [5], [21]. For the limitation data of the inclusion studies, we did not conduct a stratified analysis to investigate the effect of environmental factors such as infection status in present meta-analysis. Further studies estimating the effect of gene-environment interactions are needed to expand the knowledge of rs352140. Nonetheless, our results with accumulated data indicate that rs352140 polymorphism may play a role in cancer development, especially in Caucasians.

The SNP rs5743836, substituting tyrosine to cytosine, locates in the promoter region of *TLR9* [2]. This SNP was thought to be associated with increased transcriptional activity and function of *TLR9* [22], [47]–[50]. Noack J, et.al [24] observed that BL cell lines with C allele rs5743836 were lack of cell death upon *TLR9* triggering, suggesting that the distinct cell death responses upon CpG oligodeoxynucleotides (CpG ODN) treatment in BL cells may be linked to rs5743836. Moreover, C allele of the rs5743836 polymorphism generates an IL6 responding element. In mononuclear cells carrying the CT genotype of rs5743836, IL6 up-regulates *TLR9* expression, leading to exacerbate cellular responses to CpG, including IL6 production and B cell proliferation. In addition, previous study observed that C allele of rs5743836 create a putative NF-kB binding site and exhibits a greater NF-kB binding affinity which leads to enhanced transcription of NF-kB and results in greater release and production of pro-inflammatory mediators [49]. Hence, the presence of the C allele seems to result in enhanced NF-kB activation following *TLR9* triggering and act as an important role in host immune response, inflammation and tumorigenesis. Although some studies reveal the relationship between rs5743836 polymorphism with tumorigenesis, the evidence from epidemiological studies seems controversial. Most previous studies did not show any association between rs5743836 polymorphism and cancer risk [16], [20], [24]–[27], except three studies that performed in non-Hodgkin lymphoma from Portugal and Italy [25] and in Hodgkin’s lymphoma from Greece [22]. Our results with accumulated data did not find any significant association between rs5743836 polymorphism and overall cancer risk. In this meta-analysis, we found significant heterogeneity among studies in overall comparisons. And the frequency of minor allele in control subjects and cancer types contributes a total proportion of 62.2% to the heterogeneity. Considering the heterogeneity partly from lymphoma, we performed a sub-group analysis to investigate the effect of cancer type. Unfortunately, we did not find any evidence of a significant association between rs5743836 polymorphism and susceptibility to lymphoma. Our results were in contrast with some single study carried out from lymphoma [22], [25]. This discrepancy may be explained by two reasons: (1) genetic diversities. A very broad range of MAF was observed among the different studies which may inter individual differences to disease susceptibility. (2) exposure to different disease status. In some studies (disease), rs5743836 applies at the early stages of the neoplastic process. Other factors assume more significance in the latter stages which culminate in malignant transformation. The polymorphism may be relevant in setting the scene with induction of severe inflammation, and this may allow other factors to assume more significance later on [20], [49]. We also performed a subgroup analysis by sample size, no association between rs5743836 polymorphism and cancer risk was found. Considering significant heterogeneity in meta-analysis of rs5743836, the results should be treated with caution. Large, multiethnic and well-designed studies are needed to confirm our present results.

The SNP rs187084 located in the promoter of *TLR9*, created a putative Sp1 binding site, which may be functionally relevant [51]. Variant alleles of rs187084 polymorphisms can alter the functional ability of *TLR9* [21] and modify the response to bacterial pathogens thereby varying interindividual disease susceptibility [49]. Several studies have investigated the effect of rs187084 on human cancers, such as cervical cancer [2], [21], endometrial cancer [26], gastric cancer [18], acute lymphoblastic leukemia [16] and lymphoma [27], but most of them showed no significant associations. In the current study, the pooled examination also showed no significant association between rs187084 polymorphisms and cancer risk.

For heterogeneity, we found the frequency of minor allele in case subjects was the main source of heterogeneity for rs187084. While, the cancer types and frequency of minor allele in control subjects were the main sources of heterogeneity for rs5743836. Thus, further studies using larger numbers of participants from multiple discrete regions and sufficient cancer types should be conducted.

Some limitations of this meta-analysis should be monitored. First, most of the enrolled studies only included the association of *TLR9* polymorphisms with cancer risk and a more precise adjusted OR for other covariates like age, family history, infect condition and environmental factors were unavailable. Secondly, the studies evaluated cancer risk with rs352140 and rs187084 were limited. Hence, sub-group analysis with the specific cancer type did not performed due to insufficient data. Thirdly, for rs5743836, most participates were Caucasians. Lack of data from other ethical group may lead to an improper result. Furthermore, due to the low frequency of the minor allele, it is difficult for analysis by using any more genetic models for rs5743836. Finally, we did not found an appropriate genetic model to assess the association between rs352140 and the risk of cancer according to the Thakkinstian’s method. Thus, our results derived from genetic model of rs352140 should be debatable. In spite of these limitations, this meta-analysis with methodological quality assessment was applied and all studies included in this meta-analysis met our selection criteria clearly. Allele comparison and genetic models comparison were used to evaluate the cancer risk with TLR9 polymorphisms. Secondly, subgroups analysis by ethnicity, cancer types and sample size provided better knowledge about *TLR9* polymorphisms and cancer risk.

In conclusion, our meta-analysis indicated that *TLR9* rs352140 was associated with increased cancer risk, especially in Caucasians, suggested that polymorphisms in *TLR9* may play a role in cancer development. Since genetic diversities and cancer type were the main sources of the heterogeneity, it is critical that larger and well-designed multicenter studies based on other area populations and sufficient cancer types should be performed to re-evaluate the association. Moreover, additional future studies should combine with other potential risk factors to extend our investigations.

## Supporting Information

Table S1 **PRISMA 2009 checklist.**
(DOC)

Table S2 **Scale for methodological quality assessment.**
(DOC)

Table S3 **Multiple comparisons of genotype effects.**
(DOC)

Table S4 **Stratified analysis of the** ***TLR9*** **polymorphisms with cancer risk.**
(DOC)
