# Supplementary material for: The TLR9 Gene Polymorphisms and the Risk of Cancer: Evidence from a Meta-Analysis
Source: PLoS One. 2013 Aug 19;8(8):e71785. doi: 10.1371/journal.pone.0071785 (PMC3747197; doi:10.1371/journal.pone.0071785)
Supplement: Table S2 — Scale for methodological quality assessment. (DOC) [file pone.0071785.s002.doc]

| **Table S2. Scale for methodological quality assessment.** | |
| --- | --- |
| Criteria | Score |
| 1.Representativeness of cases |  |
| Selected from cancer registry or multiple cancer center sites | 2 |
| Selected from oncology department or cancer institute | 1 |
| Not described | 0 |
| 2.Source of controls |  |
| Population or community based | 2 |
| Hospital-based cancer-free controls | 1.5 |
| Healthy volunteers without total description | 1 |
| Cancer-free controls with related diseases | 0.5 |
| Not described | 0 |
| 3.Ascertainment of relevant cancer |  |
| Histopathologic confirmation | 2 |
| Patient medical record | 1 |
| Not described | 0 |
| 4.Sample size |  |
| >1000 | 2 |
| 200-1000 | 1 |
| <200 | 0 |
| 5.Quality control of genotyping methods |  |
| Repetition of partial/total tested samples with a different method | 1 |
| Repetition of partial/total tested samples with the same method | 0.5 |
| Not described | 0 |
