# Supplementary material for: The TLR9 Gene Polymorphisms and the Risk of Cancer: Evidence from a Meta-Analysis
Source: PLoS One. 2013 Aug 19;8(8):e71785. doi: 10.1371/journal.pone.0071785 (PMC3747197; doi:10.1371/journal.pone.0071785)
Supplement: Table S3 — Multiple comparisons of genotype effects. (DOC) [file pone.0071785.s003.doc]

**Table S3. Multiple comparisons of genotype effects.**

|  | Pooled OR examination | |  |
| --- | --- | --- | --- |
|  | OR(95%CI) | *P* | *P^a^* |
| **rs352140 C/T** |  |  |  |
| TT versus CC (OR1) | 1.414(0.854,2.342) | 0.178 | 0.078 |
| CT versus CC (OR2) | 0.937(0.746,1.178) | 0.577 | 0.181 |
| TT versus CT (OR3) | 1.309(0.996,1.721) | 0.053 | <0.01 |
| **rs187084 T/C** |  |  |  |
| CC versus TT (OR1) | 1.054( 0.857,1.296) | 0.621 | 0.237 |
| CT versus TT (OR2) | 0.992(0.784,1.255) | 0.946 | 0.082 |
| CC versus CT (OR3) | 1.008(0.797,1.276) | 0.971 | 0.271 |

OR: odds ratio; CI: confidence interval. *P*. P-value for OR; *.P*^a^. P value of Q-test for heterogeneity test.
