# Supplementary material for: The TLR9 Gene Polymorphisms and the Risk of Cancer: Evidence from a Meta-Analysis
Source: PLoS One. 2013 Aug 19;8(8):e71785. doi: 10.1371/journal.pone.0071785 (PMC3747197; doi:10.1371/journal.pone.0071785)
Supplement: Table S4 — Stratified analysis of the TLR9 polymorphisms with cancer risk. (DOC) [file pone.0071785.s004.doc]

**Table S4. Stratified analysis of the TLR9 polymorphisms with cancer risk.**

|  | N | | Case/control | Allele comparison | *P* | *P^a^* | Genetic model comparison | | *P* | *P^a^* |
| --- | --- | --- | --- | --- | --- | --- | --- | --- | --- | --- |
|  |  |  |  | OR (95% CI) |  |  |  | OR (95% CI) |  |  |
| **rs352140 C/T** | | |  |  |  |  |  |  |  |  |
| *Total* | 1616 | | 814/802 | 1.093(0.946,1.262) ^※^ | 0.228 | 0.151 | Dominant | 1.004(0.808,1.247) ^※^ | 0.973 | 0.103 |
|  |  | |  |  |  |  | Recessive | **1.320(1.019,1.709)** ^※^ | **0.035**^△^ | 0.407 |
| *Ethnicity* | | |  |  |  |  |  |  |  |  |
| Caucasian | 773 | | 403/370 | **1.263(1.029,1.551)** ^※^ | **0.026**^△^ | 0.595 | Recessive | **1.397(1.017,1.919)** ^※^ | **0.039**^△^ | 0.766 |
| Asian | 843 | | 411/432 | 0.948(0.773,1.162) ^※^ | 0.605 | 0.166 | Recessive | 1.180(0.756,1.842) ^※^ | 0.466 | 0.078 |
| **rs187084 T/C** | | |  |  |  |  |  |  |  |  |
| *Total* | 3595 | | 1743/1852 | 0.990(0.844,1.161) | 0.901 | 0.084 | Dominant | 0.982(0.849,1.241) | 0.878 | 0.063 |
|  |  | |  |  |  |  | Recessive | 1.024(0.849,1.236) ^※^ | 0.803 | 0.256 |
| *Sample size* | | | |  |  |  |  |  |  |  |
| >1000 | 2752 | | 1370/1382 | 1.077(0.967,1.2) | 0.18 | 0.64 | Recessive | 1.043(0.849,1.281) ^※^ | 0.69 | 0.556 |
| <1000 | 843 | | 373/470 | 0.828(0.568,1.207) | 0.326 | 0.096 | Recessive | 0.935(0.589,1.484) ^※^ | 0.776 | 0.055 |
| **rs5743836 T/C** | | |  |  |  |  |  |  |  |  |
| *Total* | 8880 | | 4028/4852 | 1.146(0.917,1.432) | 0.23 | <0.01 | Dominant | 1.181(0.899,1.552) | 0.233 | <0.01 |
| *Cancer type* | | |  |  |  |  |  |  |  |  |
| Lymphoma | | 6638 | 2875/3763 | 1.334(0.963,1.847) | 0.083 | <0.01 | Dominant | 1.434(0.965,2.131) | 0.075 | <0.01 |
| Other cancers | | 2242 | 1153/1089 | 0.914(0.773,1.081) | 0.292 | 0.361 | Dominant | 0.886(0.731,1.074) | 0.219 | 0.495 |
| *Sample size* | | | |  |  |  |  |  |  |  |
| >1000 | 5075 | | 2276/2799 | 1.081(0.715,1.634) | 0.714 | <0.01 | Dominant | 1.103(0.658,1.848) | 0.71 | <0.01 |
| <1000 | 3805 | | 1752/2053 | 1.188(0.890,1.587) | 0.243 | <0.01 | Dominant | 1.229(0.867,1.741) | 0.246 | <0.01 |

N. Number of comparisons; OR: odds ratio; CI: confidence interval. *P*. P-value for OR; *.P*^a^. P value of Q-test for heterogeneity test; Bold type: OR with statistical significance; △ P<0.05, considered statistically significant; ※. Fixed-effect model was used when P value for heterogeneity test >0.1.
